# Supplementary material for: Hydroxyapatite/Poly (Butylene Succinate)/Metoprolol Tartrate Composites with Controllable Drug Release and a Porous Structure for Bone Scaffold Application
Source: Polymers (Basel). 2023 Oct 24;15(21):4205. doi: 10.3390/polym15214205 (PMC10648255; doi:10.3390/polym15214205)
Supplement: Supplementary file 1 [file polymers-15-04205-s001.zip › polymers-2597379-supplementary.pdf]

Hydroxyapatite/poly (butylene succinate)/metoprolol tartrate composites with controllable drug release and porous structure for bone scaffold application

Hongming Yang[1], Rui Pan[1], Yuan Zhou[1], Guiting Liu[1]*, Rong Chen[1]*, Shaoyun Guo[1]

[1]The State Key Laboratory of Polymer Materials Engineering, Polymer Research Institute of Sichuan University, Chengdu 610065, China

*** Correspondence:**
Rong Chen；Guiting Liu
rongchen@scu.edu.cn;liugt@scu.edu.cn

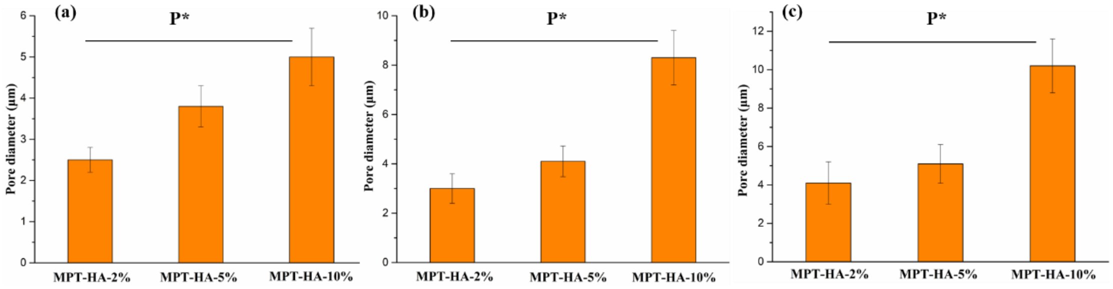

Figure S1. Pore diameter analysis of HA/PBS/MPT composites after immersion of 2 h (a), 12 h (b) and 36 h (c).

Table S1. Pore diameter (μm) of HA/PBS/MPT composites after immersion for different times

Specimen	2 h	12 h	36 h
MPT-HA-2%	2.5	3.1	4.1
MPT-HA-5%	3.8	4.2	4.9
MPT-HA-10%	5.1	8.3	10.2

The Ca/P mass ratio is 1.48, which is close to the theoretical Ca/P mass ratio (1.67) of HA.

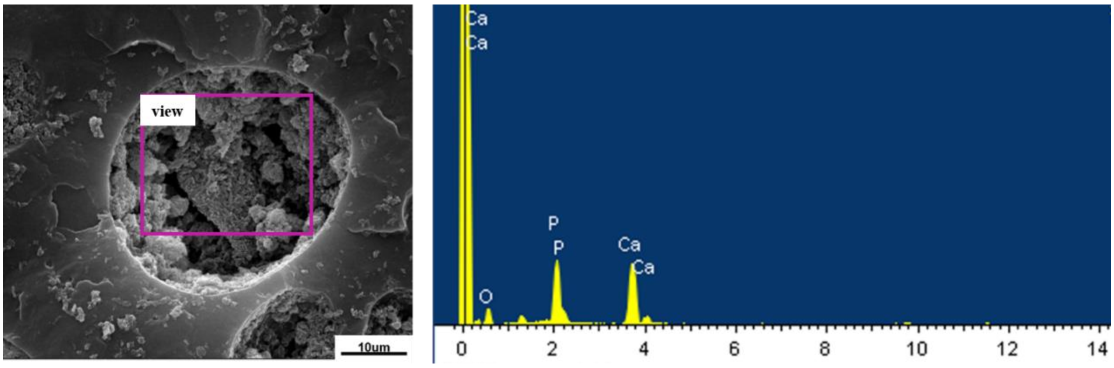

Figure S2. EDS analysis of MPT-HA-10% samples immersion in PBS for 36 hours.

Table S2. The quantitative statistics of element mass proportion

Element	Weight Percentage, %	Atomic percentage, %
O	39.16	59.14
P	23.68	16.47
Ca	37.16	24.40
